# Novelty Influences Dopamine Responses to Conditioned and Unconditioned Aversive Stimuli over Extended Temporal Windows

**DOI:** 10.1523/ENEURO.0358-25.2025

**Published:** 2025-12-04

**Authors:** Munir Gunes Kutlu, Stephanie A. Cajigas Gabriel, Jennifer Tat, Jennifer E. Zachry, Erin S. Calipari

**Affiliations:** ^1^Department of Neural Sciences, Temple University Lewis Katz School of Medicine, Philadelphia, Pennsylvania 19140; ^2^Center for Substance Abuse Research, Temple University Lewis Katz School of Medicine, Philadelphia, Pennsylvania 19140; ^3^Department of Pharmacology, Vanderbilt University, Nashville, Tennessee 37232; ^4^Vanderbilt Brain Institute, Vanderbilt University, Nashville, Tennessee 37232; ^5^Vanderbilt Center for Addiction Research, Vanderbilt University, Nashville, Tennessee 37232; ^6^Department of Molecular Physiology and Biophysics, Vanderbilt University, Nashville, Tennessee 37232; ^7^Department of Psychiatry and Behavioral Sciences, Vanderbilt University Medical Center, Nashville, Tennessee 37232

**Keywords:** aversive stimulus, dopamine, learning, novelty, nucleus accumbens, pavlovian

## Abstract

Dopamine release in the nucleus accumbens is classically linked to associative learning, signaling relationships between predictive cues and outcomes. Yet, dopamine is also strongly modulated by novelty, a nonassociative factor that has received comparatively little attention. Here, we used optical dopamine sensors in awake, behaving male and female mice to define how novelty alters the temporal dynamics of dopamine release during aversive learning. We manipulated novelty in three ways: (1) omitting expected footshocks, (2) introducing novel neutral cues concurrently with shock-predictive stimuli, and (3) presenting novel stimuli in an unpaired fashion within a context. Across all conditions, manipulations robustly increased dopamine release and in some cases altered the directionality of cue-evoked dopamine responses. Notably, these effects extended beyond the immediate stimulus window, altering subsequent responses to both conditioned cues and footshocks. Together, these findings demonstrate that changes in the environment that extend beyond prediction-based learning can exert a powerful and sustained influence on dopamine signaling, reshaping how aversive cues and outcomes are represented in the brain.

## Significance Statement

Novelty strongly shapes how organisms learn and adapt by influencing dopamine signaling in the brain. This study shows that novelty alters dopamine responses to both conditioned and unconditioned aversive stimuli, with effects that persist across short and extended timescales. These findings reveal that novelty is a key factor modulating dopamine dynamics during learning and highlight its broader role in guiding adaptive behavioral responses to changing environments.

## Introduction

Dopamine dysregulation is at the center of nearly every psychiatric disease (Davis et al., 1991; Cook et al., 1995; Di Chiara, 1999; Kaye et al., 1999; Nestler and Carlezon, 2006) and has been shown to play an important role in behavior in a wide range of contexts in response to both appetitive and aversive stimuli (Berridge and Robinson, 1998; Nicola et al., 2005; Pessiglione et al., 2006; Bromberg-Martin et al., 2010; McCutcheon et al., 2012; Stelly et al., 2019). Thus, understanding what dopamine dysregulation means for patients suffering from these complex disorders requires an understanding of how dopamine controls behavior (Davis et al., 1991; Cook et al., 1995; Kaye et al., 1999; Nestler and Carlezon, 2006). Many systems neuroscience studies have focused on the neural mechanisms of associative learning with a goal of defining how circuits in the brain encode associations between cues and predicted outcomes to control behavior. Dopamine release, especially in the nucleus accumbens (NAc) core, has been shown to play a critical causal role in this process, which has guided large bodies of work studying the role of dopamine and linking it to the ability to form associations between cues and predictive outcomes (Nicola et al., 2005; Bossert et al., 2007; Saunders et al., 2013, 2018). Within this framework, dopamine has been studied across species (Scheiner et al., 2002; Pessiglione et al., 2006; Siciliano et al., 2016), in reward-based contexts (Phillips et al., 2003; Roitman et al., 2004; Day and Carelli, 2007; Flagel et al., 2011), in response to stressful and/or aversive stimuli (Saal et al., 2003; Oleson et al., 2012; Wenzel et al., 2018; Stelly et al., 2019), and has been linked to the expression of behavior in all of these cases. However, emerging work has shown that dopamine in the NAc core is highly modulated by nonassociative factors such as novelty (how familiar a stimulus is; Berridge and Robinson, 1998; Comoli et al., 2003; Zink et al., 2004; Redgrave and Gurney, 2006; Redgrave et al., 2008; Kutlu et al., 2021, 2022). Critically, dopamine release tracks stimulus novelty to causally mediate the effects of novelty on behavior (Kutlu et al., 2022).

Associative learning, the ability to associate cues with outcomes and make predictions about what will occur in the future, is a fundamental learning process that guides adaptive behavior in nearly all contexts. In many cases, this type of learning is conceptualized as the formation of an association between a neutral stimulus that precedes the presentation of a salient stimulus, such as a reward or footshock (Rescorla and Wagner, 1972; Day and Carelli, 2007; Kutlu and Schmajuk, 2012). As these stimuli are paired repeatedly over time, the neutral stimulus becomes predictive of the outcome, acquires associative value, and elicits a conditioned behavioral response. However, this type of learning does not take place in isolation, and other environmental factors can influence the speed at which this learning occurs or the conditioned responses that these stimuli elicit. Novelty is one of these factors (Legault and Wise, 2001; Costa et al., 2014; Duszkiewicz et al., 2019). Novelty is high when a stimulus or context is encountered for the first time and decreases with repeated exposure. Both valenced and neutral stimuli exert the strongest influence on behavior when they are novel (Lubow, 1973; Hall and Channell, 1985). The introduction of novel, but irrelevant information, into an environment can reduce the conditioned response to a previously learned cue [known as external inhibition; Pavlov, 1927] even though the cue–outcome associations remain unchanged. Interestingly, dopamine release in the NAc core tracks external inhibition and habituation to valenced and neutral stimuli (Young et al., 1993; Kutlu et al., 2021, 2022). Thus, novelty is a key factor that influences dopamine release patterns in response to environmental stimuli; however, exactly how and when this occurs has not been elucidated in its entirety.

Novelty is defined by a mismatch between what is predicted and what occurs (Schmajuk et al., 1996). This can occur in an environment in multiple ways. For example, the omission of a stimulus following a predictive cue would introduce novel information into the task. This can also occur through the introduction of stimuli that have not been previously experienced but do not change anything about prior predictions (e.g., introduction of a light cue during a fear conditioning task). By modulating novelty in the environment using the examples above and recording dopamine across the tasks, we found dopamine release signatures and determined how they are influenced by different forms of novelty. We show that these forms of novelty have robust effects on dopamine release patterns that occur during the task and are maintained for long periods of time. Critically, in some cases, novelty changed the directionality of dopamine response to conditioned stimuli. Together, these data are important for a better understanding of the complexities that are involved in dopaminergic encoding of information within the NAc.

## Materials and Methods

### Animals

Male and female 6- to 8-week-old C57BL/6J mice obtained from Jackson Laboratory (SN, 000664) were kept five per cage and maintained on a 12 h reverse light/dark cycle with *ad libitum* access to food and water. Experiments were conducted in accordance with the guidelines of the Institutional Animal Care and Use Committee at Vanderbilt University School of Medicine. The order of testing was counterbalanced, and experimenters were blind to experimental groups throughout behavioral experiments.

### Surgical procedure

At least 1 h prior to surgery, mice were administered ketoprofen (5 mg/kg) via subcutaneous injection. Mice were anesthetized using isoflurane (5% for induction and 2% for maintenance) and placed on a stereotaxic frame (David Kopf Instruments) as described previously (Kutlu et al., 2020, 2021). Using a 0.10 ml NanoFil syringe (World Precision Instruments) with a 34 gauge needle, AAV5.CAG.dLight1.1 (University of California, Irvine; Patriarchi et al., 2018) was unilaterally infused into the NAc (bregma coordinates: anterior/posterior, +1.4 mm; medial/lateral, +1.5 mm; dorsal/ventral, −4.3 mm; 10° angle) at a rate of 50 nl/min for a total volume of 500 nl. Following infusion, the needle was kept at the injection site for 7 min before being slowly withdrawn. Fiber-optic cannulas (400 μm core diameter; 0.48 NA; Doric Lenses) were then implanted in the NAc and positioned immediately dorsal to the viral injection site (bregma coordinates, anterior/posterior, +1.4 mm; medial/lateral, +1.5 mm; dorsal/ventral, −4.2 mm; 10° angle) before being permanently fixed to the skull using adhesive cement (C&B Metabond; Parkell). Mice were allowed a minimum of 6 weeks to recover to ensure efficient viral expression before commencing experiments.

### Histology

Mice were deeply anesthetized with an intraperitoneal injection of a ketamine/xylazine mixture (100 mg/kg; 10 mg/kg) before being transcardially perfused with 10 ml of 1× PBS solution followed by 10 ml of cold 4% PFA in 1× PBS. Animals were subsequently decapitated, and the brain was extracted and postfixed in the 4% PFA solution stored at 4°C for at least 48 h before being dehydrated in a 30% sucrose in 1× PBS solution stored at 4°C. After sinking, the tissue was sectioned (35 μm slices) on a freezing sliding microtome (Leica SM2010R; Leica Biosystems) and then placed in a cryoprotectant solution (7.5% sucrose + 15% ethylene glycol in 0.1 M PB) stored at −20°C until immunohistochemical processing. Sections were mounted on glass microscope slides with ProLong Gold antifade reagent. Fluorescent imaging was conducted using a BZ-X700 inverted fluorescence microscope (Keyence) under a dry 20× objective (Nikon). Injection site locations and optical fiber placements were determined with serial images in all experimental animals (Extended Data Fig. 1-2).

### Fiber photometry

The fiber photometry system used two light-emitting diodes (LEDs; 490 and 405 nm; Thorlabs) controlled by an LED driver (Thorlabs). The 490 nm light source was filtered with a 470 nm (the excitation peak of dLight1.1) bandpass filter and the 405 nm light source was used as an isosbestic control (Patriarchi et al., 2018). Light was passed through an optical fiber (400 μm, 0.48 NA; Doric Lenses) that was coupled to a chronically implanted fiber-optic cannula in each mouse. LEDs were controlled via a real-time signal processor (RZ5P; Tucker-Davis Technologies), and emission signals from each LED stimulation were determined by multiplexing. The Synapse software (Tucker-Davis Technologies) was used to control the timing and intensity of the LEDs and to record the emitted fluorescent signals upon detection by a photoreceiver (Newport Visible Femtowatt Photoreceiver Module; Doric Lenses). LED power (125 μW) was measured daily and maintained across trials and experiments. For each event of interest (e.g., cue presentation, footshock), transistor–transistor logic (TTL) signals were used to timestamp onset times from the Med-PC V software (Med Associates) and were detected via the RZ5P in the Synapse software (see below). A built-in low–pass filter on the Synapse software was set to 10 Hz to eliminate noise in the fiber photometry raw data.

### Behavioral experiments

Mice were trained and tested daily in individual operant conditioning chambers (Med Associates) fitted with visual and auditory stimuli, including a standard house light, a white noise generator, and a 16-tone generator capable of outputting frequencies between 1 and 20 kHz (85 dB).

#### Footshock omission

**Figure 1.**
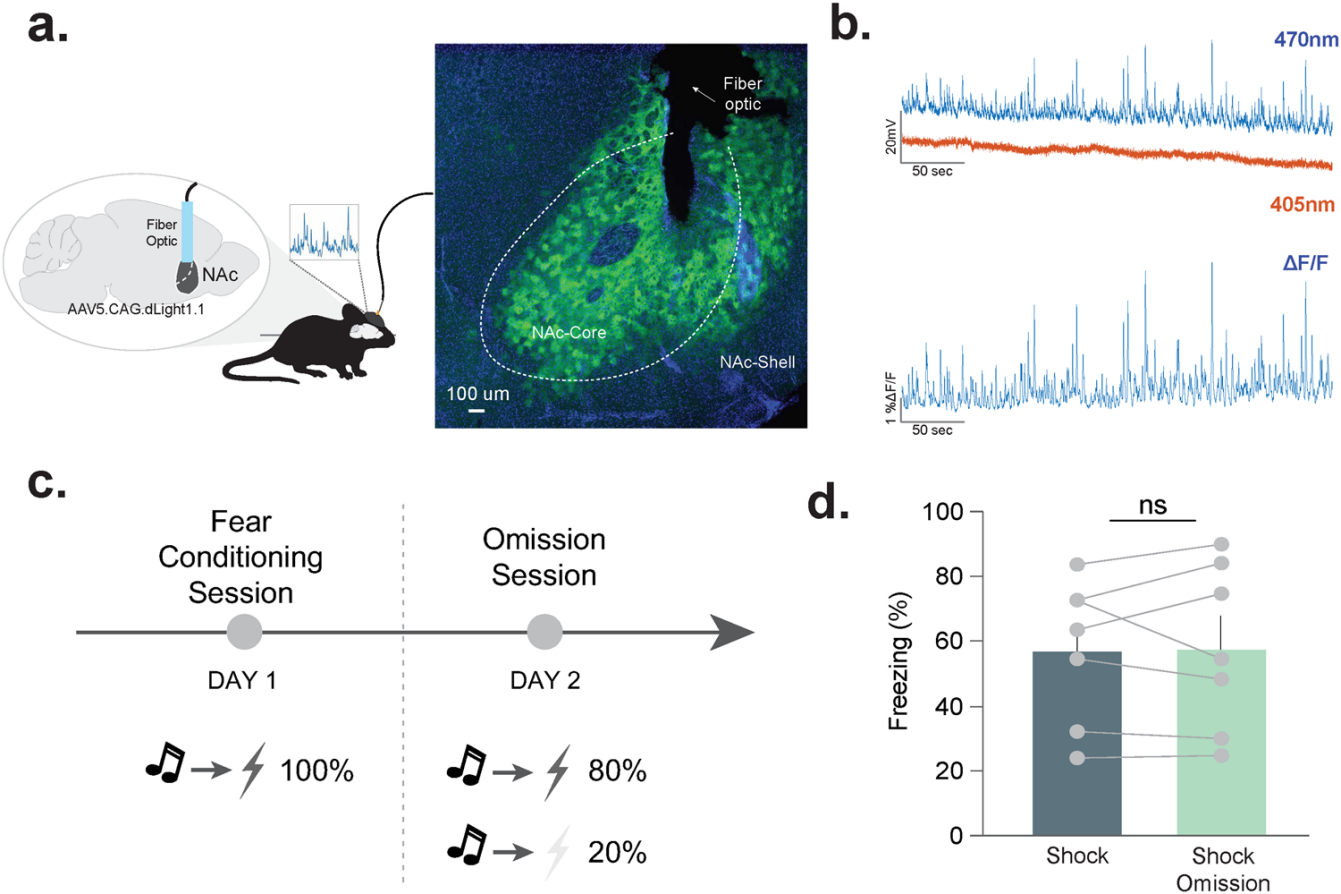
Experimental design: unexpected footshock omission. ***a***, Schematic of viral surgery approach and representative histology confirming viral expression and fiber-optic placement in the NAc core (see Extended Data Figs. 1-1, 1-2 for analysis approach and viral placement). ***b***, Representative fiber photometry recordings. Experimental (470 nm) and control (405 nm) channels (top) with Δ*F*/*F* normalization (bottom). ***c***, Design of fear conditioning experiments with training on Day 1 and the omission session on Day 2. ***d***, The percentage of time spent freezing did not differ across training and testing days or between the footshock presentation and omission trials (paired *t* test; *t*_(6)_ = 0.11; *p* = 0.91; *n* = 7 mice). Data represented as mean ± SEM.

**Figure 2.**
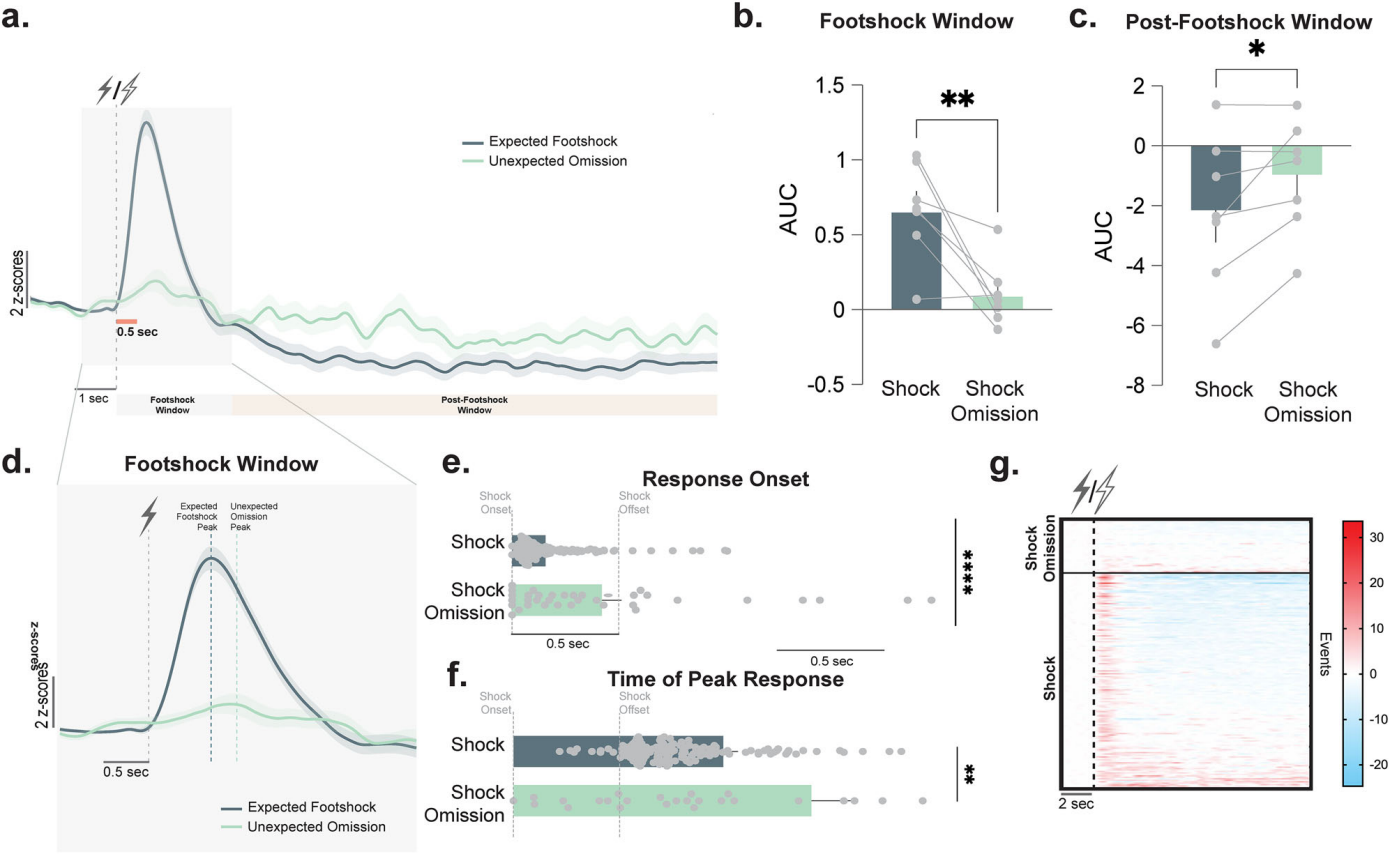
Unexpected footshock omission increased dopamine at the time of omitted shock as well as over longer periods. ***a***, Averaged dopamine recordings at the time of the expected shock or unexpected shock omission in the same animals. ***b***, AUC during the footshock window (denoted in Fig. 2*a*) was smaller when the shock was omitted (paired *t* test; *t*_(6)_ = 3.78; *p* = 0.009; *n* = 7 mice). ***c***, AUC determined during the postfootshock window denoted in Figure 2*a*. The AUC was larger in trials where an unexpected omission occurred (paired *t* test; *t*_(6)_ = 2.55; *p* = 0.04; *n* = 7 mice). ***d***, The dopamine response during the footshock window for each trial type. The footshock was presented at the first dotted line and lasted for 0.5 s (noted by the solid line denoting this time on the *x*-axis). Dotted lines for each trial type denote the peak of the averaged traces across trials. ***e***, The time at which the dopamine response to the shock (or during omission) commenced was calculated as time in seconds following the footshock presentation where the dopamine transient reached 5% of its eventual peak (or 0.5 *z*-score above their baseline; unpaired *t* test; *t*_(181)_ = 5.05; *p* < 0.0001; *n* = 36–147 events). This was lower during an expected footshock, indicating that the onset of the signal occurred faster. ***f***, Time in the trial following the footshock presentation, where the dopamine response reached its peak. This was increased on trials where the footshock was omitted, indicating the signal peaked at a later point in the trial (unpaired *t* test; *t*_(181)_ = 2.748; *p* = 0.0066; *n* = 36–147 events). ***g***, Heatmaps of all trials across all animals (each line is an individual trial in a single animal). Omission trials are at the top, and footshock trials are at the bottom. See Extended Data Fig. 2-1 for additional analysis. Data are represented as mean ± SEM. #*p* = 0.07; *p < 0.05; **p < 0.01; ****p < 0.0001.

Mice underwent a single conditioning session (Day 1) of pavlovian fear conditioning wherein they received a 5 s auditory cue (tone; 85 dB, 2.5 kHz frequency) which was immediately followed by a footshock (1.0 mA, 0.5 s duration) for 11 pairings with a variable interstimulus interval (40–90 s). On Day 2, 80% of the trials were the same as Day 1; however, on the other 20% of trials, the cue was presented, but the footshock was omitted (total of 21 shock trials and 5–6 omission trials). Each session—for this experiment as well as those following—was recorded using cameras, and the videos were scored to determine freezing behavior, identified as the time of immobility except respiration during the stimulus duration, was calculated and converted into percent freezing [(freezing time * 100) / (stimulus duration)]. This was done for this series of experiments as well as each of the following experiments (Figs. 1, 2).

#### Introduction of a novel cue during fear conditioning

**Figure 3.**
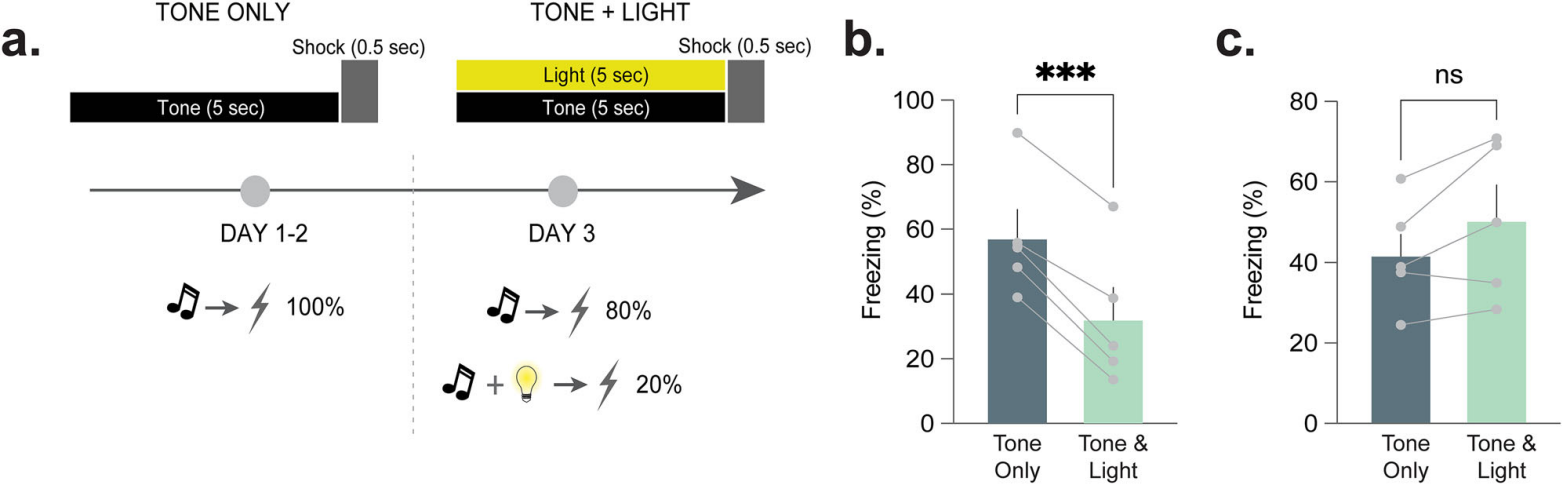
Experimental design: presentation of a novel and neutral stimulus during fear conditioning. ***a***, Mice were trained for 2 d in a fear conditioning task where a cue predicted the presentation of a brief footshock. On the third day, all parameters remained the same, except that a novel, neutral stimulus was presented concurrently with the previously learned fear cue on 20% of trials (tone + light), and the remaining 80% were the fear cue only (tone only). ***b***, Freezing decreased when an unexpected stimulus (house light) was presented with tone on Day 3 compared with tone-only trials during the same day, displaying an external inibition effect (paired *t* test; *t*_(4)_ = 10.39; *p* = 0.0005; *n* = 5 mice). ***c***, Mice that did not display an external inhibition effect had no change in freezing response between tone-only and tone + light trials (paired *t* test; *t*_(4)_ = 2.23; *p* = 0.09; *n* = 5 mice). Data are represented as mean ± SEM. ***p < 0.001.

**Figure 4.**
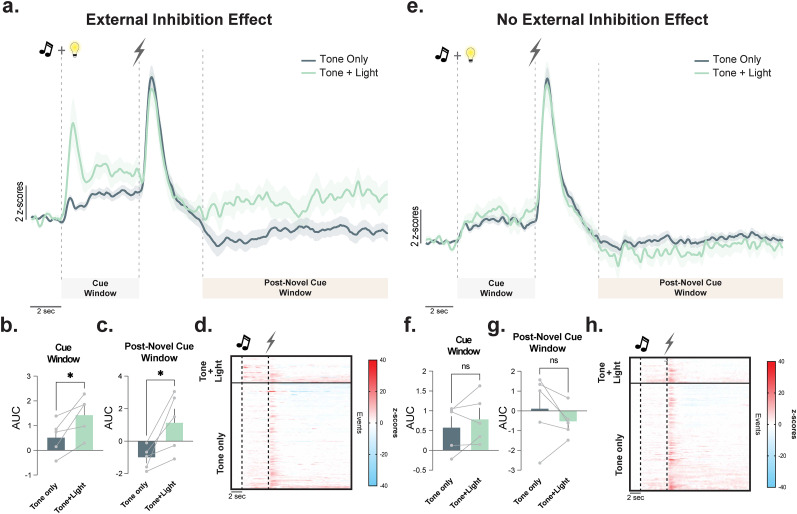
Dopamine release patterns to novel, neutral stimulus differ based on behavioral displays of external inhibition. ***a***, Averaged dopamine recordings by the trial type for the tone-only (fear cue by itself) and the tone + light (fear cue with novel neutral cue) trials from the mice that showed external inhibition. This trace includes the cue response, the shock response, and a post-response window that shows dopamine levels over an extended period. ***b***, AUC at the time of the cue presentation was significantly larger when the novel stimulus (light) was presented in conjunction with the previously learned tone (paired *t* test; *t*_(4)_ = 3.22; *p* = 0.032; *n* = 5 mice). ***c***, AUC during the post-novel cue presentation was significantly larger when the novel stimulus (light) was presented in conjunction with the previously learned tone (paired *t* test; *t*_(4)_ = 3.19; *p* = 0.033; *n* = 5 mice). ***d***, Heatmaps of all trials across all animals (each line is an individual trial in a single animal). Tone + light (fear cue with a novel neutral cue) are at the top and tone-only trials (fear cue) are at the bottom. ***e***, Averaged dopamine responses across the entire trial for each trial type (tone only, tone + light) from the mice that did not show external inhibition. This trace includes the cue response, the shock response, and a post-response window that shows dopamine levels over an extended period. ***f***, AUC at the time of the cue presentation was no different when a novel stimulus (light) was presented in conjunction with the previously learned tone compared with tone only (paired *t* test; *t*_(4)_ = 1.21; *p* = 0.29; *n* = 5 mice). ***g***, AUC at the time of the cue presentation was no different when a novel stimulus (light) was presented in conjunction with the previously learned tone compared with tone only (paired *t* test; *t*_(4)_ = 1.25; *p* = 0.27; *n* = 5 mice). ***h***, Heatmaps of all trials across all animals (each line is an individual trial in a single animal). Tone + light (fear cue with a novel neutral cue) are at the top, and tone-only trials (fear cue) are at the bottom. Data are represented as mean ± SEM. *p < 0.05.

Mice underwent 2 d of fear conditioning as described above, where 100% of the trials resulted in an auditory cue (tone) followed by a footshock (11 trials for each day). On Day 3, all tone presentations were followed immediately by a footshock, like the first two conditioning sessions; however, on 20% of the trials, an unexpected novel cue (house light) was introduced simultaneously with the tone that predicted shock (20–21 cue–shock and 5–6 cue–light–shock trials). To determine whether an animal exhibited external inhibition, we averaged freezing responses across all tone–shock trials and compared them with freezing during tone–light–shock trials. Animals were classified as showing external inhibition if freezing was greater on tone–shock than on tone–light–shock trials and as “no external inhibition” otherwise (Figs. 3, 4).

#### Dishabituation

**Figure 5.**
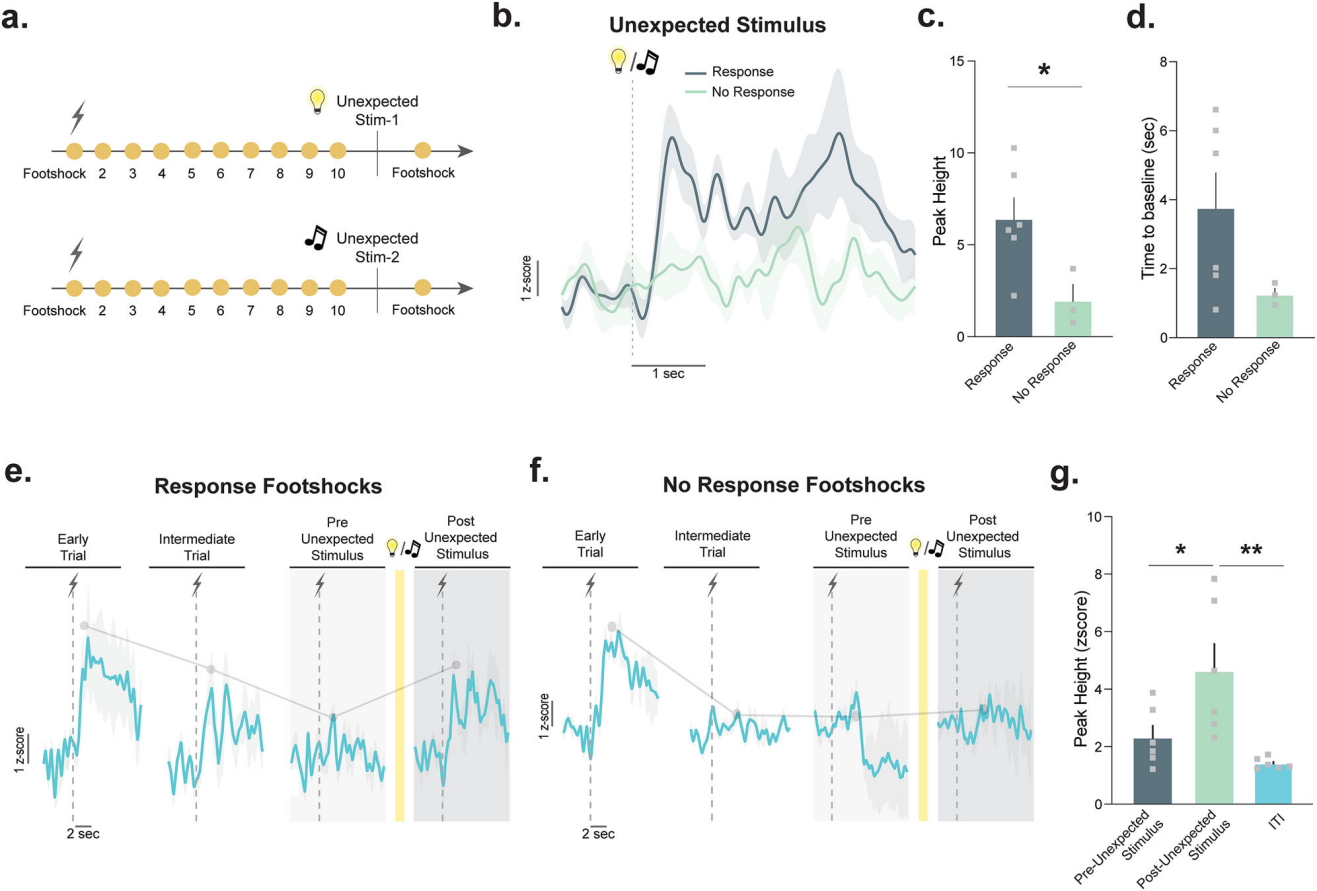
Unexpected novelty presented in an unpaired fashion increases dopamine response to a subsequent footshock. ***a***, Timeline of unexpected stimulus presentation. ***b***, Averaged dopamine response to the unexpected stimulus for the trials that exhibited a behavioral response to the unexpected stimulus (response) versus trials that the mice showed no behavioral response (no response; see Extended Data Fig. 5-1 for dopamine responses in animals that did not show an orienting response). ***c***, Peak dopamine signal following the unexpected stimulus was significantly greater in the trials that exhibited a behavioral response to the unexpected stimulus (unpaired *t* test; *t*_(7)_ = 2.50; *p* = 0.04; *n* = 3–6 mice). ***d***, Time for the dopamine response to return to baseline following peak (unpaired *t* test; *t*_(7)_ = 1.67; *p* = 0.1372; *n* = 3–6 mice). ***e***, In a single session, the mice exhibited a behavioral response to repeated footshocks, as indicated by the dopamine signal. ***f***, Single session dopamine signal to repeated footshocks in the group that did not exhibit a behavioral response to the unexpected stimulus. ***g***, Data from the response trials (no-response trial data presented in Extended Data Fig. 5-2). The response had a larger shock-evoked dopamine response following the novel stimulus compared with the trial before the novel stimulus or the dopamine response during the ITI period [ANOVA, *F*_(2,15)_ = 7.61; *p* = 0.0052; Tukey’s post hocs (preunexpected stim. vs postunexpected stim. *p* = 0.0391; preunexpected stim. vs ITI *p* = 0.555; postunexpected stim. vs ITI *p* = 0.0048); *n* = 6 mice]. Data are represented as mean ± SEM. *p < 0.05; **p < 0.01.

Mice were presented with a total of 32 footshocks (1 mA, 0.5 s duration) during a single session. Each footshock was separated by a 15 s intershock interval. Prior to the 11th and 22nd footshocks, a stimulus (house light or tone, 85 dB, 2.5 kHz frequency) was presented for a 3 s duration in the middle of the preceding intershock interval. Both stimuli were presented in individual sessions, and the order of their presentation was counterbalanced between animals. We reviewed all behavioral videos for the dishabituation experiments to make sure unexpected stimulus presentations resulted in a behavioral response to ensure that mice attended to the novel stimulus. The behavioral responses were identified as an orienting response (i.e., turning head toward the light or speakers) or sudden movement at the stimulus onset. We identified three trials where animals did not exhibit any behavioral signature (orienting response for one trial was not determined because the view of the camera was blocked). The dopamine response data from these trials were analyzed separately as “no response” as a control (Fig. 5).

### Data analysis

The analysis of the fiber photometry data was conducted using a custom MATLAB pipeline, as we have described previously (Kutlu et al., 2021, 2022). Raw 470 nm (F470 channel) and isosbestic 405 nm (F405 channel) traces were collected at a rate of 1,000 samples per second (1 kHz). The raw data from each individual channel (450 or 470 nM) were then minimally filtered using a lowess filter before calculating Δ*f*/*f* values via polynomial curve fitting. For the lowess filter, the number of data points for calculating the filtered value was set to 0.0004 (values closer to 1 indicate stronger filtering). Δ*f*/*f* for the entire trace was calculated as (F470 − F405 nm) / F405 nm. This transformation uses the isosbestic F405 nm channel, which is not responsive to fluctuations in calcium, to control for calcium-independent fluctuations in the signal and to control for photobleaching. For analysis, data were cropped around behavioral events using TTL pulses, and for each experiment, 2 s of pre-TTL up to 20 s of post-TTL Δ*F*/*F* values were analyzed. *Z*-scores were calculated by taking the pre-TTL Δ*F*/*F* values as the baseline (*z*-score = (TTLsignal − b_mean) / b_stdev, where TTL signal is the Δ*F*/*F* value for each post-TTL time point, b_mean is the baseline mean, and b_stdev is the baseline standard deviation). This allowed for the determination of dopamine events that occurred at the precise moment of each significant behavioral event.

For statistical analysis, we calculated the AUC, peak height, time to the baseline, tau, and *R*^2^ values for each individual dopamine event (Yorgason et al., 2011; see Extended Data Fig. 1-1 for the visual description of these values). The AUCs were calculated via trapezoidal numerical integration on each of the *z*-scores across a fixed timescale. The peak height values were the maximum values after the TTL onset. The time to the baseline was computed as the seconds to going back to the 0 *z*-score baseline, and tau was the duration to the two-third of the peak height. For both measures where individual curves did not reach the baseline or tau, the minimum value was taken into the statistical analysis. For slope analysis, *R*^2^ values for the fitted curves were computed (linear polynomial curve) for a 15 s duration. Finally, the time to threshold was calculated as the time point where each dopamine response reached 5% of its peak or 0.5 *z*-score above its baseline. The duration of the data collection for the AUC, peak height, time to the baseline, and tau values was determined by limiting the analysis to the *z*-scores between 0 time point (TTL signal onset) and the time where the dopamine peak of interest returns to the baseline. Unpaired *t* tests were employed to test the group differences for all fiber photometry-based dependent variables. We also calculated maximum *z*-scores for event fiber photometry traces and analyzed to see if these were significantly different from the critical *z*-score at *p* = 0.05 level (1.645) using independent *t* tests. Alpha was 0.05 for all statistical analyses. Statistical analyses were performed using GraphPad Prism (version 8; GraphPad Software) and MATLAB (MathWorks). All data were depicted as group mean ± standard error of the mean (SEM).

## Results

### Omission of an expected footshock increases dopamine over extended periods of time

Dopamine release was recorded using fiber photometry and the optical dopamine sensor dLight 1.1 expressed in the NAc core (Fig. 1; see also Extended Data Fig. 1-2). For this experiment, fear conditioning was employed. On the first training day, mice received 11 tone–footshock pairings (Fig. 1*c*). On the second day, footshock was omitted for 20% of the trials. Importantly, freezing to the tone did not change between days; thus, differences in the behavioral responses that preceded unexpected omissions cannot explain any differences in dopamine responses (Fig. 1*d*; paired *t* test; *t*_(6)_ = 0.11; *p* = 0.99; *n* = 7 mice).

10.1523/ENEURO.0358-25.2025.f1-1 Figure 1-1 **Illustrated dopamine peak.** Dopamine peak illustrated with metrics of interest for time locked events and calculations such as area under the curve, peak height, time to baseline and tau. Download Figure 1-1, TIF file.

10.1523/ENEURO.0358-25.2025.f1-2 Figure 1-2 **Schematic detailing optic implant placement.** The fiber photometry **i**mplants were placed in the core of the medial nucleus accumbens. Left, placements for fiber photometry studies, N = 13. Download Figure 1-2, TIF file.

A positive dopamine transient was evoked by both the presentation of an expected footshock as well as at the time of the expected but omitted footshock (Fig. 2*a–c*,*g*). These responses differed in terms of the magnitude of the area under the curve (AUC) of the dopamine transient as well (Fig. 2*b*; paired *t* test; *t*_(6)_ = 3.78; *p* = 0.009; *n* = 7 mice; also see Extended Data Fig. 2-1*a* for the comparison to the ITI baseline). The dopamine response to the footshock occurred very rapidly following shock onset—indicating that the dopamine response occurred to the footshock itself rather than in response to the shock offset (Fig. 2*d–f*). In omission trials, the onset of the dopamine response (Fig. 2*c*,*e*; unpaired *t* test; *t*_(181)_ = 5.05; *p* < 0.0001; *n* = 36–147 events) and the peak of this response were delayed (Fig. 2*c*,*f*; unpaired *t* test; *t*_(181)_ = 2.748; *p* = 0.0061; *n* = 36–147 events) in comparison with trials where the footshock was present.

10.1523/ENEURO.0358-25.2025.f2-1 Figure 2-1 **Dopamine responses to shock omission and external inhibition predict freezing behavior. (a)** The dopamine response to shock omission was significantly larger than the dopamine release during the inter-trial interval (ITI). Area under the curve (AUC) during the footshock window (denoted in Fig. 2a) was smaller when the shock was omitted; however, the omission-induced dopamine response was still larger than the dopamine response at baseline (ITI) (RM ANOVA, F(1.123,6.739) = 20.06, p = 0.0028, Holm-Sidak post-hocs [Shock vs. Omission p = 0.0182; Shock vs. ITI p = 0.0049; Omission vs. ITI p = 0.0295]). **(b)** The decrease in freezing response during external inhibition was negatively correlated with dopamine release, such that the dopamine response elicited by the addition of the unexpected light stimulus was inversely related to the drop in freezing (r = -0.3997; p = 0.0389). **(c)** Moreover, complete abolition of freezing was associated with significantly increased dopamine release: external inhibition (Tone + Light) trials without freezing exhibited higher dopamine levels compared to trials with freezing (unpaired t-test; t(25) = 3.56, p = 0.0015; n = 16 freezing trials vs. 11 no freezing trials). Data are represented as mean ± S.E.M. * p < 0.05, ** p < 0.01. Download Figure 2-1, TIF file.

Importantly, the unexpected shock omission had a lasting effect on the dopamine signal that occurred after the omission and into the postfootshock window (Fig. 2*a*,*g*). There was a larger response (measured as AUC)—indicating a higher dopamine baseline—as compared with the trials where the footshock was presented as expected (Fig. 2*c*; paired *t* test; *t*_(6)_ = 2.55; *p* = 0.04; *n* = 7 mice, five males and two females). Thus, when unexpected information is encountered, the dopamine patterns differ in a manner that extends beyond simple stimulus-specific coding. We hypothesized that this signal was driven by novelty being introduced in the form of an omission in this case. Thus, novelty in any form should be able to induce a similar signature, which we tested subsequently.

### Increasing novelty via the presentation of a novel, neutral stimulus alters behavior-dependent, task-related dopamine responses

Mice underwent fear conditioning as described above for two sessions (Days 1 and 2) wherein a tone predicted the presentation of a footshock, but no other stimuli were presented (Fig. 3*a*). On the third session (Day 3), an unexpected house light (novel cue) was presented in conjunction with the previously learned fear tone for 20% of the trials. Freezing decreased in a subset of mice in response to the unexpected light (Fig. 3*b*; paired *t* test; *t*_(5)_ = 10.39; *p* = 0.0005; *n* = 5 mice, four males and one female), but not in another set of mice (Fig. 3*c*; paired *t* test; *t*_(5)_ = 2.23; *p* = 0.09; *n* = 5 mice, three males and two females). The ability of an unexpected neutral stimulus (house light) to interfere with freezing behavior (i.e., reduce freezing) has been reported widely in the literature and is called external inhibition (Pavlov, 1927; Wenger, 1936; Gagné, 1941).

During the trials where the unexpected stimulus was presented, dopamine response to both the fear cue alone and the fear cue + the novel cue was positive in mice exhibiting an external inhibition effect (Day 3; Fig. 4*a*,*d*). The peak dopamine response when the novel cue was presented, represented as AUC, was increased as compared with trials where the fear cue was presented alone (Fig. 4*b*; paired *t* test; *t*_(4)_ = 3.19; *p* = 0.032; *n* = 5 mice), and time to return to the baseline represented as postfootshock window AUC was also increased (Fig. 4*c*; paired *t* test; *t*_(4)_ = 3.22; *p* = 0.033; *n* = 5 mice) indicating that the response was larger and slower to decay when novelty was introduced. Finally, the dopamine response during the introduction of the unexpected light stimulus was negatively correlated with freezing response (Extended Data Fig. 2-1*b*,*c*). However, mice that did not exhibit an external inhibition effect did not have the same dopamine release signatures. Mice that did not display a change in freezing with the introduction of a novel, unexpected neutral stimulus did not have the observed long temporal signal (Fig. 4*e*,*h*). Dopamine release during both the cue and post novel cue windows was not different between tone-only and tone + light presentations (Fig. 4*f*; paired *t* test; *t*_(4)_ = 1.21; *p* = 0.29; *n* = 5 mice; Fig. 4*g*; paired *t* test; *t*_(4)_ = 1.25; *p* = 0.27; *n* = 5 mice, respectively). These data are also interesting as in cases where no novelty is presented, we and others have shown that fear cues in this region result in decreases in dopamine release in response to the conditioned cue (Oleson et al., 2012; Stelly et al., 2019; Kutlu et al., 2021, 2022). Here, dopamine responses to fear cues are positive when the environment includes novelty, and this positive response is even larger during the precise time of novelty presentation. Thus, the introduction of novelty can change the directionality of task-specific dopamine responses.

### The introduction of an unpaired novel, neutral stimulus increases footshock-evoked dopamine responses in subsequent trials

Ten footshocks were presented with a constant interstimulus interval (15 s; Fig. 5*a*). Following the tenth footshock presentation, mice received an unexpected presentation of an auditory or a visual stimulus (tone or light) during the interstimulus interval. To ensure the inclusion of the trials wherein animals noticed the unexpected stimulus presentation, we separately analyzed the trials in which animals exhibited a behavioral response to the unexpected stimulus (orienting response or a movement response at the onset of the unexpected stimulus; response trials, *n* = 6) and trials in which animals showed no behavioral response (no-response trials; *n* = 3). We found that unexpected stimulus presentations coupled with a behavioral response resulted in a positive dopamine response, whereas unexpected stimulus presentations that did not result in a behavioral response did not induce a significant dopamine response (Fig. 5*b*). The peak of the dopamine response was increased for the unexpected novel and neutral stimulus during its presentation when animals oriented to the stimulus (Fig. 5*c* unpaired *t* test; *t*_(7)_ = 2.50; *p* = 0.04; *n* = 7 mice; five males and two females) as compared with when they did not (Extended Data Fig. 5-1*b*). The time to the baseline did not reach significance between the two response types (Fig. 5*d*; unpaired *t* test; *t*_(7)_ = 1.67; *p* = 1.67).

10.1523/ENEURO.0358-25.2025.f5-1 Figure 5-1 Unexpected stimulus: Dopamine responses in mice showing no orienting response. **(a)** Dopamine signal pre- and post-unexpected stimulus presentation was unchanged. **(b)** Peak dopamine signal (unpaired t-test, t(4)=0.067, p=0.9493), **(c)** time for dopamine to return to baseline following peak (unpaired t-test, t(4)=0.691, p=0.5275), and **(d)** tau (unpaired t-test, t(4)=1.430, p=0.2259) did not differ between pre- and post-unexpected trials. Data represented as mean ± S.E.M. Download Figure 5-1, TIF file.

10.1523/ENEURO.0358-25.2025.f5-2 Figure 5-2 Dopamine responses to footshocks across the disinhibition paradigm. **(a)** Dishabituation in Response trials. Trial-by-trial dopamine response to footshocks (10 trials pre-unexpected stimulus and 1 trial post-unexpected stimulus) for trials where animals exhibited a behavioral response to the unexpected stimulus (Response). **(b)** Failed dishabituation in No Response trials. Trial-by-trial dopamine response to footshocks (10 trials pre-unexpected stimulus and 1 trial post-unexpected stimulus) for trials that animals did not show behavioral responses to the unexpected stimulus. Download Figure 5-2, TIF file.

Following the unexpected stimulus presentation, the dopamine response elicited by a footshock increased following the response trials (Fig. 5*e*,*g*; ANOVA, *F*_(2,15)_ = 7.61; *p* = 0.0052; Tukey's post hocs; preunexpected stim. vs postunexpected stim. *p* = 0.0391; preunexpected stim. vs ITI *p* = 0.555; postunexpected stim. vs ITI *p* = 0.0048) but not following the no-response trials (Fig. 5*f*; see Extended Data Fig. 5-2 for the values for the no-response trials) compared with the preunexpected stimulus footshock dopamine response (Extended Data Fig. 5-2). Following the response trials, the peak of the dopamine response to the footshock was significantly higher postunexpected stimulus compared with the preunexpected stimulus footshock and the dopamine response during the ITI period (Fig. 5*g*). Together, these data highlight the long-lasting effects of novelty on dopamine release dynamics and demonstrate that presentation of an unexpected stimulus—even outside of defined trials—leads to increase in the dopamine response to subsequent stimulus presentations.

## Discussion

Models of dopamine function have traditionally emphasized its role in associative learning, where phasic signals encode prediction errors that update cue–outcome associations (Schultz et al., 1997; Nicola et al., 2005; Day and Carelli, 2007; Flagel et al., 2011). While this framework has been influential, it does not fully capture all of the contributions of dopamine to behavioral control (Berridge and Robinson, 1998; Bromberg-Martin et al., 2010). For example, many models that explain dopamine in this way are unable to account for behavioral phenomena controlled by novelty (Sutton and Barto, 1990; Schultz et al., 1997; e.g., latent inhibition, habituation to neutral stimuli, changes in behavior in response to novelty). Our data demonstrate that novelty exerts a robust and sustained influence on dopamine release in the NAc core. Dopamine release is increased by (1) altering the presentation of predicted stimuli, (2) introducing neutral and novel stimuli concurrently with a previously learned predictive cue, and (3) introducing a novel stimulus in an unpaired fashion. All of these manipulations altered how dopamine responded to not only the novel stimulus but also other stimuli in the environment. Furthermore, this impact extends beyond the immediate window of stimulus presentation, which changes how neural systems respond to information in an environment to control behavior.

The introduction of novelty robustly changed dopamine release patterns in the NAc core in response to previously learned stimuli—including their directionality. Previous work has shown that aversive cues in isolation elicit reductions in dopamine in the NAc core (Oleson et al., 2012; Stelly et al., 2019; Kutlu et al., 2021, 2022). This effect has been repeated over different types of approaches and in many labs, including our own (Oleson et al., 2012; Stelly et al., 2019; Kutlu et al., 2021, 2022). While some of these studies have used analytical approaches to record dopamine, even work using optical dopamine sensors in the NAc core from our group and others have observed reductions in dopamine responses to fear cues when they are presented in isolation (Badrinarayan et al., 2012; McCutcheon et al., 2012; Oleson et al., 2012; de Jong et al., 2019; Stelly et al., 2019; Kutlu et al., 2021, 2022). However, in the present study, when novel stimuli were presented concurrently with shock-predictive cues, dopamine responses increased. Interestingly, even on the trials where the fear cue was presented alone, this decrease was no longer observed. Thus, the simple addition of a novel light cue into the environment can change the directionality of dopamine responses to predictive cues. Indeed, previous work has shown that in the presence of novel light cues, dopamine responses to fear cues were increased (Kutlu et al., 2022). These data show the role of novelty in shaping dopamine response patterns that influence stimulus processing over long periods of time and highlight the complex role that novelty can play in shaping the relationship between neural signals and behavioral variables.

Notably, these increases in dopamine release were prolonged and independent of novelty type (omission or addition of stimuli in an environment), highlighting a complex role novelty plays in the relationship between neural signals and behavior. Prior work has focused on the immediate effects of novelty on dopamine release patterns, where increasing novelty increases dopamine release while dopamine release decreases as a stimulus becomes more familiar over repeated exposure (i.e., less novel; Joseph et al., 1993, 2000; Young et al., 1993, 2005; Morrens et al., 2020; Kutlu et al., 2022). In addition to the immediate effects of novelty on dopamine release patterns, following any novelty, dopamine baselines were increased for an extended duration of time (up to ∼20 s). Prior studies employing methods with low temporal resolution have also revealed novelty-induced potentiation of dopamine release (Joseph et al., 1993; Young et al., 1993), which might be the result of the sustained dopamine responses observed in the current study.

These long-lasting dopamine responses could also explain why novelty, even when not explicitly paired with a predictive cue, can alter subsequent stimulus processing. Behavioral theories of novelty have proposed that increased novelty in the environment biases attention and modulates learning about temporally adjacent events in the same context (Schmajuk et al., 1996; Kutlu and Schmajuk, 2012). Consistent with this, prior studies have shown that unexpected changes enhance orienting responses (Hall and Channell, 1985; Swan and Pearce, 1988) and disrupt latent inhibition, a novelty-driven learning phenomenon in which pre-exposure to a stimulus normally reduces subsequent conditioning (Hall and Channell, 1985; Joseph et al., 2000; Young et al., 2005). In our experiments, repeated footshock presentations led to habituated dopamine responses, but the unexpected insertion of a novel stimulus amplified the dopamine response to subsequent shocks. Thus, novel stimuli in an environment are capable of influencing dopamine responses to other familiar stimuli, even when they are not experienced concurrently.

Several key questions remain for future work. One is how novelty influences dopamine levels over extended timescales. It is unclear whether novelty actively increases dopamine signaling over longer time periods or instead prevents the gradual reductions that typically occur with habituation. Both interpretations converge on the same principle—that dopamine levels remain elevated when the environment is novel—but they imply different underlying mechanisms of regulation. A second question is how these studies relate to traditional error-based models of learning where omissions in reward-based outcomes result in transient reductions in dopamine (observed in the pauses in dopamine neuron firing in the midbrain at reward omission; Schultz et al., 1997). This may reflect the fact that dopamine represents multiple, sometimes competing, informational variables (Engelhard et al., 2019)—including novelty (Kutlu et al., 2022), salience (Kutlu et al., 2021), and prediction-based algorithms (Schultz et al., 1997; Jeong et al., 2022)—and that novelty may dynamically reshape how these signals are integrated within accumbens circuits. Disentangling these overlapping contributions will be an important direction for future studies.

Dopamine reliably tracks the presence of novelty. In the NAc core, novelty consistently produced increases in dopamine release, revealing a unidirectional effect that reshaped dopamine responses to both conditioned and unconditioned stimuli. These data highlight the complexity of neural encoding in dynamic environments. Stimuli rarely occur in isolation, and novelty provides a powerful signal that influences how other stimuli and events are processed. This capacity for novelty to dynamically reconfigure dopamine responses is likely to support flexible behavioral adaptation when things change unexpectedly. Understanding how novelty interacts with prediction, salience, and valence will therefore be essential for building a comprehensive account of dopamine’s role in behavior and its dysregulation in disease.

## Synthesis

Reviewing Editor: Christie Fowler, University of California Irvine Decisions are customarily a result of the Reviewing Editor and the peer reviewers coming together and discussing their recommendations until a consensus is reached. When revisions are invited, a fact-based synthesis statement explaining their decision and outlining what is needed to prepare a revision will be listed below. The following reviewer(s) agreed to reveal their identity: NONE. Note: If this manuscript was transferred from JNeurosci and a decision was made to accept the manuscript without peer review, a synthesis may not be available.

This manuscript was reviewed by two experts in the field. Both appreciated the findings, noting that the work advances the field by building on prior work by this group and others showing the important role novelty plays in shaping both the dopamine response and behavior during learning and fear conditioning. It shows how dopamine to footshock is modulated not only by the shock itself, but by unexpected / novel paired and unpaired stimuli in the environment during the task. It is critical to understand the role of dopamine in tasks that extend beyond studies that are performed in a tightly controlled behavioral vacuum that is likely not representative of most (if not all) real work settings. This study builds on these models by adding novel and unexpected environmental stimuli to probe how dopamine function and informs behavior in complex environments. Specific comments are as follows:

1. The findings are overstated in the significance statement related to providing a framework for novelty processing in psychiatric disease. Please rewrite.

2. Was an orienting response confirmed in Figure 3 (Tone + Light) studies in mice that did not display external inhibition (Figure 3C). This confirmation was performed in the unexpected stimulus task in Figure 5 and modified the dopamine response. Perhaps the no external inhibition animals did notice the novel light, which doesn't undermine (rather may support) the conclusions.

3. Classic RPE studies show a "dip" in dopamine following reward omission, which is presumably novel, at least in early trials. Is reward different from fear conditioning with respect to the role of novelty on dopamine response? Or perhaps RPE dopamine dips from omission only occur after several trials when novelty fades? Related, is the bump in dopamine to greater than expected outcomes really novelty? Although speculative, some more discussion might be interesting on novelty in classic RPE data.

4. The dopamine post-novel cue are interesting. Tone+Light is indeed larger here as indicated by authors. Is Tone only dopamine being suppressed in this window as the reason driving the difference. The authors argue it is Tone+Light activation rather than Tone alone suppression.

Minor:

p.3, line 100: 'valenced'

p.4, line 118: 'within the NAc'

p.7, lines 180-181: 'cue was presented by the footshock was omitted' ?

Fig 1g legend: shock omission trials are at the top of this panel

## Author Response

eN-TNWR-0358-25X. We were excited to see the enthusiasm from the reviewers about the impact and quality of our manuscript. The comments note some spaces where the manuscript can be clarified and improved, and we have addressed all of the comments either in the manuscript text or in our responses below. Manuscript changes are noted with highlighted text.

Synthesis statement: (Comments for the author) This manuscript was reviewed by two experts in the field. Both appreciated the findings, noting that the work advances the field by building on prior work by this group and others showing the important role novelty plays in shaping both the dopamine response and behavior during learning and fear conditioning. It shows how dopamine to footshock is modulated not only by the shock itself, but by unexpected / novel paired and unpaired stimuli in the environment during the task. It is critical to understand the role of dopamine in tasks that extend beyond studies that are performed in a tightly controlled behavioral vacuum that is likely not representative of most (if not all) real work settings. This study builds on these models by adding novel and unexpected environmental stimuli to probe how dopamine function and informs behavior in complex environments.

Response: We thank the reviewers for the succinct and accurate summary of our work and their enthusiasm for its broader implications. We agree there is more to do to refine our conclusions, and we have revised the manuscript accordingly as detailed below.

1. The findings are overstated in the significance statement related to providing a framework for novelty processing in psychiatric disease. Please rewrite.

Response: We have revised our statement to more directly assess the work as contained within the manuscript. It now reads as follows: "Novelty strongly shapes how organisms learn and adapt by influencing dopamine signaling in the brain. This study shows that novelty alters dopamine responses to both conditioned and unconditioned aversive stimuli, with effects that persist across short and extended timescales. These findings reveal that novelty is a key factor modulating dopamine dynamics during learning and highlight its broader role in guiding adaptive behavioral responses to changing environments" This revision removes the previous overstatement regarding psychiatric disease while maintaining the broader relevance of our findings.

2. Was an orienting response confirmed in Figure 3 (Tone + Light) studies in mice that did not display external inhibition (Figure 3C). This confirmation was performed in the unexpected stimulus task in Figure 5 and modified the dopamine response. Perhaps the no external inhibition animals did notice the novel light, which doesn't undermine (rather may support) the conclusions.

Response: This is an interesting comment. While we agree that assessing orienting behavior would provide valuable complementary information, the behavioral videos collected for these experiments were optimized for automated freezing detection rather than fine-grained posture or head-movement analyses. The camera position, frame rate, and lighting were sufficient to quantify freezing with high reliability but not to resolve the subtle, transient head or body reorientations that characterize orienting responses. Accurately scoring those movements requires a different camera angle and higher temporal and spatial resolution. Therefore, we cannot definitively confirm orienting behavior in these sessions. However, given that similar stimuli reliably elicit orienting in other tasks using comparable parameters, it is likely that the mice did detect the novel light even if this was not quantifiable here. Future experiments designed with high-resolution behavioral tracking will be necessary to quantify orienting directly.

3. Classic RPE studies show a "dip" in dopamine following reward omission, which is presumably novel, at least in early trials. Is reward different from fear conditioning with respect to the role of novelty on dopamine response? Or perhaps RPE dopamine dips from omission only occur after several trials when novelty fades? Related, is the bump in dopamine to greater than expected outcomes really novelty? Although speculative, some more discussion might be interesting on novelty in classic RPE data.

Response: We appreciate this thoughtful comment. Classic reward prediction error (RPE) studies indeed report transient decreases in dopamine neuron firing following reward omission. However, those effects are typically measured in midbrain dopamine neurons and may not always translate directly to dopamine release in terminal regions such as the nucleus accumbens, where robust local regulation can shape release independently of firing. Moreover, our study focuses on aversive learning, where the dynamics and valence of dopamine signaling differ from reward paradigms. It is possible that novelty modulates these systems differently - enhancing dopamine release when predictions are violated, independent of outcome valence. We have expanded the Discussion to include this comparison and to note how novelty may contribute to apparent asymmetries between classic RPE models and the sustained dopamine increases observed here. Below is the text that addresses this concern and comment 4 that was added to the discussion: "Several key questions remain for future work. One is how novelty influences dopamine levels over extended timescales. It is unclear whether novelty actively increases dopamine signaling over longer time periods or instead prevents the gradual reductions that typically occur with habituation. Both interpretations converge on the same principle - that dopamine levels remain elevated when the environment is novel - but they imply different underlying mechanisms of regulation. A second question is how these studies relate to traditional error-based models of learning where omissions in reward-based outcomes result in transient reductions in dopamine (observed in the pauses in dopamine neuron firing in the midbrain at reward omission). This may reflect the fact that dopamine represents multiple, sometimes competing, informational variables - including novelty, salience, and prediction-based algorithms - and that novelty may dynamically reshape how these signals are integrated within accumbens circuits. Disentangling these overlapping contributions will be an important goal for future studies." 4. The dopamine post-novel cue are interesting. Tone+Light is indeed larger here as indicated by authors. Is Tone only dopamine being suppressed in this window as the reason driving the difference. The authors argue it is Tone+Light activation rather than Tone alone suppression.

Response: We want to thank the reviewers for this opportunity to enhance our discussion. This is an interesting point, whether the tone+light increases dopamine during long windows or whether tone only decreases. The point that we are trying to make is that when novel stimuli are encountered that dopamine levels are different from when novelty is not there over long periods, but it is possible that when predictions are repeatedly met that dopamine slowly is reduced and that novelty is simply increasing levels back to the initial baseline. In this framework, tonic dopamine would send the novelty in the environment and slowly change background levels to report this. Both of these situations are equally possible and get at the same idea - that novelty changes dopamine levels in the NAc on long time scales. It is unclear from these studies exactly how this happens, but we have now discussed this in the manuscript and future work will focus on understanding how this occurs. The text that addresses this is above in the response to comment 3.

5. Minor corrections: p.3, line 100: 'valenced' ; p.4, line 118: 'within the NAc' ; p.7, lines 180-181: 'cue was presented by the footshock was omitted' ? ; Fig 1g legend: shock omission trials are at the top of this panel Response: We corrected these mistakes in the manuscript.
