# Supplementary figures and images for: Novelty Influences Dopamine Responses to Conditioned and Unconditioned Aversive Stimuli over Extended Temporal Windows
Source: eNeuro. 2025 Dec 4;12(12):ENEURO.0358-25.2025. doi: 10.1523/ENEURO.0358-25.2025 (PMC12685013; doi:10.1523/ENEURO.0358-25.2025)

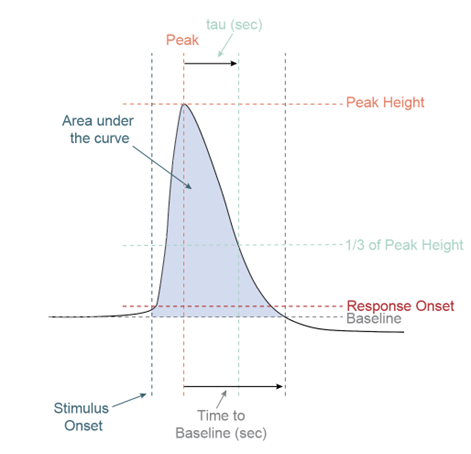

Supplement: Figure 1-1 — Illustrated dopamine peak. Dopamine peak illustrated with metrics of interest for time locked events and calculations such as area under the curve, peak height, time to baseline and tau. Download Figure 1-1, TIF file. [file eneuro-12-ENEURO.0358-25.2025-s001.tif]

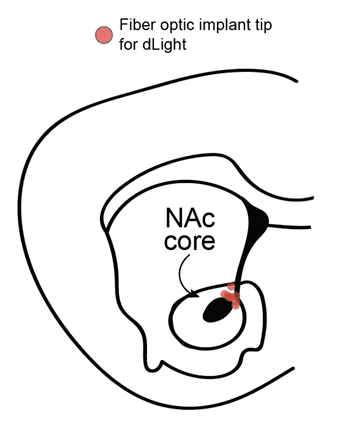

Supplement: Figure 1-2 — Schematic detailing optic implant placement. The fiber photometry implants were placed in the core of the medial nucleus accumbens. Left, placements for fiber photometry studies, N = 13. Download Figure 1-2, TIF file. [file eneuro-12-ENEURO.0358-25.2025-s002.tif]

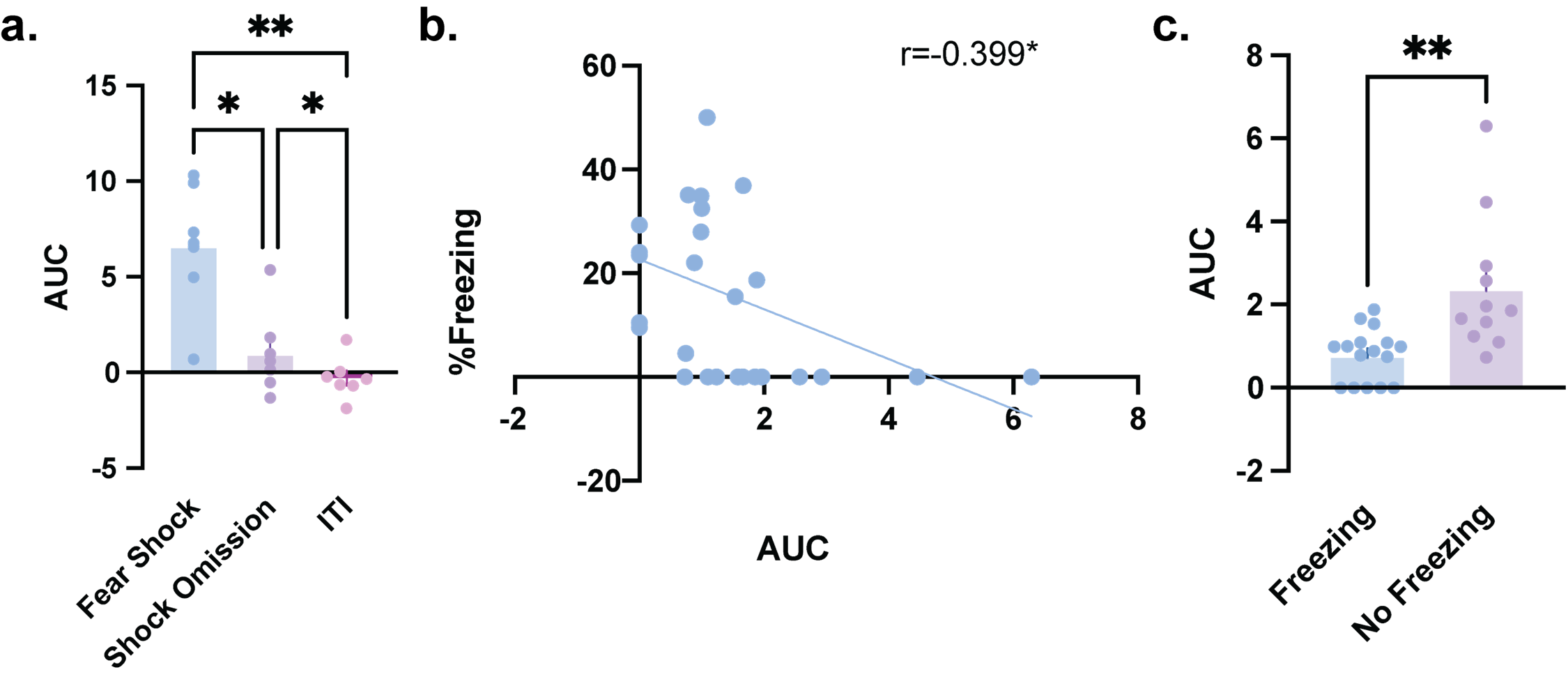

Supplement: Figure 2-1 — Dopamine responses to shock omission and external inhibition predict freezing behavior. (a) The dopamine response to shock omission was significantly larger than the dopamine release during the inter-trial interval (ITI). Area under the curve (AUC) during the footshock window (denoted in Fig. 2a) was smaller when the shock was omitted; however, the omission-induced dopamine response was still larger than the dopamine response at baseline (ITI) (RM ANOVA, F(1.123,6.739) = 20.06, p = 0.0028, Holm-Sidak post-hocs [Shock vs. Omission p = 0.0182; Shock vs. ITI p = 0.0049; Omission vs. ITI p = 0.0295]). (b) The decrease in freezing response during external inhibition was negatively correlated with dopamine release, such that the dopamine response elicited by the addition of the unexpected light stimulus was inversely related to the drop in freezing (r = -0.3997; p = 0.0389). (c) Moreover, complete abolition of freezing was associated with significantly increased dopamine release: external inhibition (Tone + Light) trials without freezing exhibited higher dopamine levels compared to trials with freezing (unpaired t-test; t(25) = 3.56, p = 0.0015; n = 16 freezing trials vs. 11 no freezing trials). Data are represented as mean ± S.E.M. * p < 0.05, ** p < 0.01. Download Figure 2-1, TIF file. [file eneuro-12-ENEURO.0358-25.2025-s003.tif]

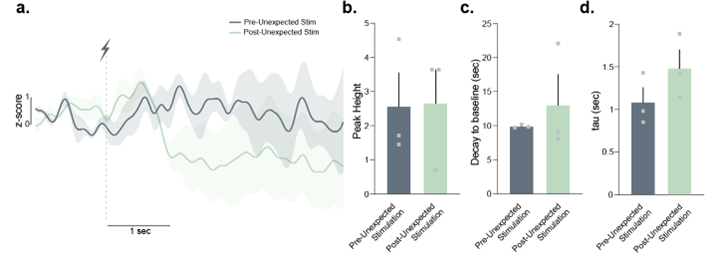

Supplement: Figure 5-1 — Unexpected stimulus: Dopamine responses in mice showing no orienting response. (a) Dopamine signal pre- and post-unexpected stimulus presentation was unchanged. (b) Peak dopamine signal (unpaired t-test, t(4)=0.067, p=0.9493), (c) time for dopamine to return to baseline following peak (unpaired t-test, t(4)=0.691, p=0.5275), and (d) tau (unpaired t-test, t(4)=1.430, p=0.2259) did not differ between pre- and post-unexpected trials. Data represented as mean ± S.E.M. Download Figure 5-1, TIF file. [file eneuro-12-ENEURO.0358-25.2025-s004.tif]

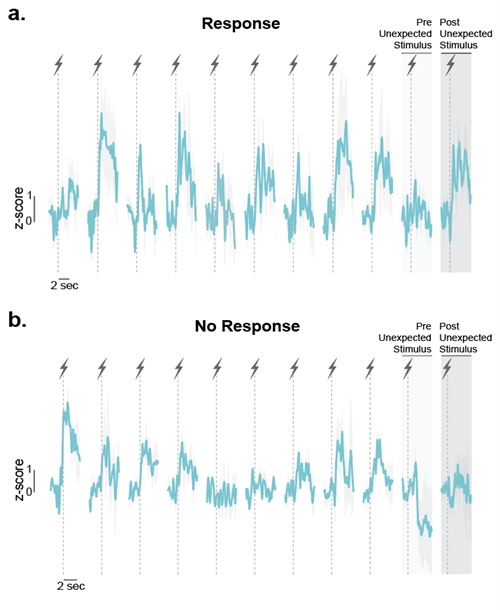

Supplement: Figure 5-2 — Dopamine responses to footshocks across the disinhibition paradigm. (a) Dishabituation in Response trials. Trial-by-trial dopamine response to footshocks (10 trials pre-unexpected stimulus and 1 trial post-unexpected stimulus) for trials where animals exhibited a behavioral response to the unexpected stimulus (Response). (b) Failed dishabituation in No Response trials. Trial-by-trial dopamine response to footshocks (10 trials pre-unexpected stimulus and 1 trial post-unexpected stimulus) for trials that animals did not show behavioral responses to the unexpected stimulus. Download Figure 5-2, TIF file. [file eneuro-12-ENEURO.0358-25.2025-s005.tif]
